# Clinical predictors of recovery of COVID-19 associated-abnormal liver function test 2 months after hospital discharge

**DOI:** 10.1038/s41598-022-22741-9

**Published:** 2022-10-26

**Authors:** Justin Y. Lu, Scott L. Ho, Alexandra Buczek, Roman Fleysher, Wei Hou, Kristina Chacko, Tim Q. Duong

**Affiliations:** 1 Department of Radiology, Albert Einstein College of Medicine and Montefiore Medical Center, 111 E 210Th St, Bronx, NY 10467 USA; 2 Department of Family, Population and Preventive Medicine, Stony Brook Medicine, Stony Brook, NY USA; 3 Division of Hepatology, Department of Medicine, Albert Einstein College of Medicine and Montefiore Medical Center, Bronx, NY USA

**Keywords:** Hepatocytes, Liver

## Abstract

This study investigated whether acute liver injury (ALI) persisted and identified predictors of ALI recovery [as indicated by alanine aminotransferase (ALT) level] at hospital discharge and 2 months post-discharge for 7595 hospitalized COVID-19 patients from the Montefiore Health System (03/11/2020–06/03/2021). Mild liver injury (mLI) was defined as ALT = 1.5–5 ULN, and severe livery injury (sLI) was ALT ≥ 5 ULN. Logistic regression was used to identify predictors of ALI onset and recovery. There were 4571 (60.2%), 2306 (30.4%), 718 (9.5%) patients with no liver injury (nLI), mLI and sLI, respectively. Males showed higher incidence of sLI and mLI (p < 0.05). Mortality odds ratio was 4.15 [95% CI 3.41, 5.05, p < 0.001] for sLI and 1.69 [95% CI 1.47, 1.96, p < 0.001] for mLI compared to nLI. The top predictors (ALT, lactate dehydrogenase, ferritin, lymphocytes) accurately predicted sLI onset up to three days prior. Only 33.5% of mLI and 17.1% of sLI patients (survivors) recovered completely at hospital discharge. Most ALI patients (76.7–82.4%) recovered completely ~ 2 months post-discharge. The top predictors accurately predicted recovery post discharge with 83.2 ± 2.2% accuracy. In conclusion, most COVID-19 patients with ALI recovered completely ~ 2 months post discharge. Early identification of patients at-risk of persistent ALI could help to prevent long-term liver complications.

## Introduction

Acute livery injury (ALI), a significant complication of coronavirus disease 2019 (COVID-19)^1,2^, has been associated with elevated risk of critical illness and mortality^3–10^. The virus responsible for COVID-19, severe acute respiratory syndrome coronavirus 2 (SARS-CoV-2), could directly infect liver cells via angiotensin-converting enzyme 2 (ACE2) receptors but evidence of direct infection is controversial^11–13^. Systemic hypoxia, sepsis, disproportional host-immune responses, and hepatotoxicity from COVID-19 treatments could also contribute to ALI in COVID-19 indirectly^14–19^. Although ALI associated with COVID-19 has been documented^3–10^, it is unknown whether COVID-19 ALI is transient or persistent. It is important to identify early on which COVID-19 patients with ALI are at risk of developing persistent liver injury to enable follow-up care to prevent long-term liver dysfunction and complications.

The goal of this study was thus to investigate whether ALI persisted in COVID-19 patients and to identify predictors of ALI recovery at hospital discharge and post hospital discharge. COVID-19 patients were stratified by no, mild and severe liver injury (nLI, mLI and sLI, respectively) based on alanine aminotransferase (ALT) level. We analyzed demographic data, comorbidities, vital signs, and laboratory tests at ALI diagnosis, hospital discharge and 2 months post discharge of patients in the Montefiore Health System in the Bronx environs in New York City. Predictive models were used to identify the top predictors of ALI onset and ALI recovery.

## Methods

### Study design, population and data source

This retrospective study was approved by the Einstein-Montefiore Institutional Review Board (#2020-11389) with an exemption for informed consent and a HIPAA waiver and was performed in accordance with relevant guidelines and regulations. The Montefiore Health System is one of the largest healthcare systems in New York City with 15 hospitals located in the Bronx, the lower Hudson Valley, and Westchester County serving a large, low-income, and racially and ethnically diverse population that was hit hard by COVID-19 early in the pandemic^20–22^.

**Figure 1:**
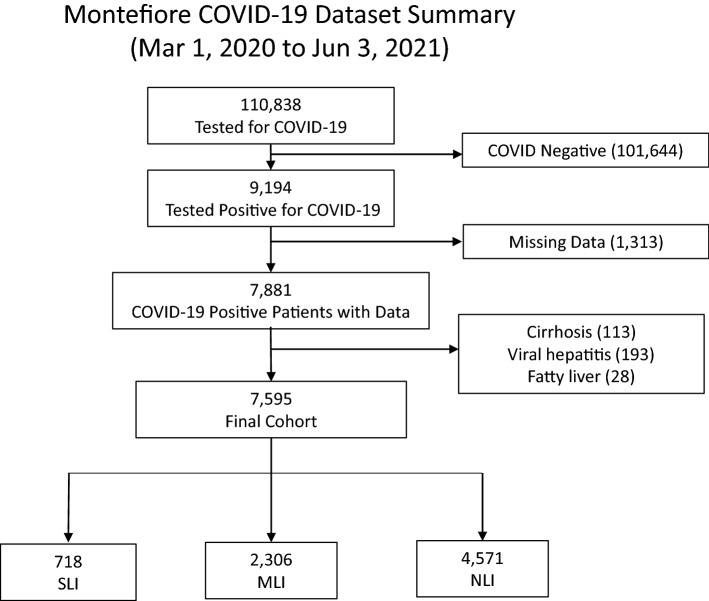
Flowchart of hospitalized patient selection. From March 11, 2020 to June 3, 2021, there were a total of 110,838 hospitalized patients had tests for COVID-19 and 9194 had a positive COVID-19 test.

From March 11, 2020 to June 3, 2021 (Fig. 1), there were 9194 hospitalized COVID-19 patients (defined by PCR test). Patients missing alanine aminotransferase (ALT) were excluded. Patients identified to have pre-existing liver diseases, including viral hepatitis, alcohol and non-alcohol related fatty liver disease and cirrhosis were excluded, as the aim of the study was to evaluate the natural history of COVID-related ALI and these patients typically would have abnormal baseline liver tests. The final sample size was 7595 hospitalized COVID-19 patients.

The data used in this study were searched and extracted as described previously^20–22^. De-identified data were made available by the Montefiore Einstein Center for Health Data Innovations after standardization to the Observational Medical Outcomes Partnership (OMOP) Common Data Model (CDM) version 6. OMOP CDM stores the health data, which comes from many sources, into standard vocabulary concepts^20–22^. This facilitates the systematic analysis of different observational databases, which includes data from the electronic medical record system, disease classification systems and administrative claims such as SNOWMED, ICD-10, LOINC, etc. Vocabulary concepts were then searched by ATLAS, a web-based tool that allows for navigation of patient-level, observational data in the CDM format developed by the Observational Health Data Sciences and Informatics (OHDSI) community, in order to build the cohort of patients. DB Browser for SQLite (version 3.12.0) was used to export and query data as SQLite database files.

Demographic data (e.g. age, sex, ethnicity, race), chronic comorbidities [e.g. congestive heart failure (CHF), chronic kidney disease (CKD), obesity, diabetes, chronic obstructive pulmonary disease (COPD), asthma] and longitudinal laboratory tests [e.g. ALT, aspartate aminotransferase (AST), alkaline phosphatase (ALP), bilirubin, ferritin (FERR), lactate dehydrogenase (LDH), brain natriuretic peptide (BNP), d-dimer (DDIM), lymphocyte count, prothrombin time (PT)], and vital signs (e.g. oximetry, temperature, systolic and diastolic blood pressure, heart rate, respiratory rate) were extracted. These laboratory values were obtained at within a day of COVID-19 hospital admission, at discharge (or closest to discharge), and 51 days (median) post hospital discharge. Steroid and remdesivir used to treat COVID-19, invasive mechanical ventilation (IMV) and ICU admission status were also extracted.

### Definitions of ALI onset and recovery

Patients were divided into 3 groups based on liver injury (LI) defined by ALT level with ULN of 30 U/L: (i) no liver injury (nLI) with ALT < 1.5 ULN [45 U/L], (ii) mild liver injury (mLI) with ALT = 1.5–5 ULN and (iii) severe livery injury (sLI) with ALT ≥ 5 ULN. Patients who had mLI but later had sLI during hospitalization were placed in the sLI group.

Complete recovery from liver injury was defined as ALT dropped below the absolute threshold of < 1.5 ULN [< 45 U/L]. Partial recovery was defined as ALT dropped below 50% of its peak value for individual patients. Recovery was assessed at hospital discharge for COVID-19 survivors only and post hospital discharge of COVID-19 survivors who returned to our health system.

### Temporal profiles of clinical variables

Although AST, ALT, ALP and total bilirubin are commonly used tests to assess liver injury and often correlated, we chose to time lock to ALT for the onset because ALT is liver specific compared the other liver function biomarkers^23–25^ (see “Discussion”). Temporal progression of clinical data was time-locked to outcome and compared among groups (nLI, mLI, sLI). Laboratory test variables were plotted across time with time lock (t = 0) to ALI onset. Data were plotted 3 days prior to and 3 days after ALI onset. For comparison, time series data for no ALI patients were time locked (t = 0) to 3 days after ED admission, along with data three days before and three days after that time point.

### Prediction of sLI onset and recovery

Logistic regression was used to rank the importance of clinical variables and predict sLI onset and recovery at each day prior to the outcome. For predicting sLI onset, clinical variables prior to sLI were used. For predicting recovery at 2-month post discharge, clinical variables at hospital discharge were used. Ranking was performed using all clinical variables with logistic regression and prediction performance was evaluated using the area under the curve (AUC) of the receiver operating characteristic (ROC). For the combined prediction models, AST was excluded from AUC analysis due to its strong correlation with ALT.

### Statistical analysis

All statistical analyses were performed using Python packages Sklearn and Statsmodels and SAS. Group differences in frequencies and percentages for categorical variables were tested using χ^2^ or Fisher’s exact tests, as deemed appropriate. Age, expressed as median (interquartile range, IQR), were compared between groups using analysis of variance (ANOVA) and post-hoc pairwise t-tests. In addition to unadjusted mortality rate, adjusted odds ratios (ORs) with 95% confidence intervals (CI) for mortality were estimated for each group (with NI as the reference group) in logistic regression adjusted for respective significant covariates. Means of clinical variables in time series graphs were analyzed via linear mixed model and least-squares means. Kaplan–Meier recovery curves for sLI and mLI after onset during hospitalization as defined by absolute threshold were plotted. p-values < 0.05 were considered statistically significant and corrected for multiple comparison using the Bonferroni method.

### Consent to participate

Consent was waived as this retrospective study was approved by the Einstein-Montefiore Institutional Review Board (#2020-11389) with an exemption for informed consent and a HIPAA waiver.

## Results

### Liver injury onset

#### Demographics and comorbidities

Table 1 summarizes the sample sizes, demographics and comorbidities for the nLI, mLI and sLI patients. There were 4571 (60.2%), 2306 (30.4%), 718 (9.5%) patients with nLI, mLI and sLI, respectively. sLI patients were significantly younger (61 ± 16.4 yo) than mLI patients (62.8 ± 16.3 yo) who in turn were also younger than nLI (65.6 ± 18.5 yo) (p < 0.05 for all pair-wise comparisons). mLI and sLI groups had markedly fewer females (37% and 34%, respectively) compared to nLI (51% females, p < 0.05), and fewer Black and more Hispanic patients compared to nLI (p < 0.01). Obesity, diabetes, congestive heart failure, chronic kidney disease, hypertension, coronary artery disease, COPD and asthma rates were significantly lower in the sLI and mLI compared to the nLI group (p < 0.05), consistent with age-related comorbidities.

**Table 1:** Demographics, comorbidities, and laboratory variables at admission of nLI, mLI, and sLI groups. Group comparison of categorical variables in frequencies and percentages used Chi-squared test or Fisher exact tests. Group comparison of continuous variables in means and SEMs (standard error of means) used the Mann–Whitney U test. All values are in n (%) unless otherwise specified.

	nLI	mLI	sLI	nLI vs mLI	nLI vs sLI	mLI vs sLI
N (%)	4571 (60.2%)	2306 (30.4%)	718 (9.5%)			
**Demographics**						
Age in years, mean (sd)	65.6 ± 18.5	62.8 ± 16.3	61 ± 16.4	*	#	$
Female gender, n (%)	2532 (55.4%)	883 (38.3%)	249 (34.7%)	*	#	
**Race/ethnicity, n (%)**						
Black/African American	1594 (34.9%)	671 (29.1%)	204 (28.4%)		#	$
White	486 (10.6%)	210 (9.1%)	62 (8.6%)			
Other	646 (14.1%)	427 (18.5%)	143 (19.9%)		#	$
Hispanic	1845 (40.4%)	998 (43.3%)	309 (43.0%)			
**Comorbidities, n (%)**						
Hypertension	1551 (33.9%)	610 (26.5%)	197 (27.4%)	*	#	
Diabetes	1511 (33.1%)	607 (26.3%)	206 (28.7%)	*	#	
Congestive heart failure	304 (6.7%)	87 (3.8%)	32 (4.5%)	*		
COPD	516 (11.3%)	184 (8.0%)	54 (7.5%)	*	#	
Coronary artery disease	435 (9.5%)	159 (6.9%)	50 (7.0%)	*		
Chronic kidney disease	1511 (33.1%)	607 (26.3%)	206 (28.7%)	*		
Obesity	552 (12.1%)	238 (10.3%)	77 (10.7%)			
Invasive mechanical ventilation	888 (19.4%)	260 (11.3%)	139 (19.4%)	*		$
ICU	1332 (29.1%)	646 (28.0%)	331 (46.1%)		#	$
Steroid	1687 (36.9%)	1283 (55.6%)	453 (63.1%)	*	#	$
Remdesivir	1002 (21.9%)	709 (30.7%)	184 (25.6%)	*		$
In hospital mortality	704 (15.4%)	494 (21.4%)	275 (38.3%)	*	#	$

### Treatments

Steroids were used most frequently in the sLI group followed by mLI and nLI (p < 0.05 for all pair-wise comparisons). Remdesivir therapy was significantly more utilized in the sLI group compared to mLI and nLI group (p < 0.05). Use of IMV was not significantly different across groups (p > 0.05).

### In-hospital mortality

The unadjusted mortality rate was significantly higher in sLI, group compared to the mLI group which was higher than the nLI group (38.3%, 21.4% and 15.4%, respectively, p < 0.05 for all pair-wise comparisons). After adjusting for significant covariates between groups, sLI patients had 4.15 times higher odds of death compared to nLI patients [95% CI 3.41, 5.05, p < 0.001], mLI had 1.69 times higher odds of death compared to nLI patients [95% CI 1.47, 1.96, p < 0.001], and sLI patients had 2.66 times higher odds of death compared to mLI patients [95% CI 2.17, 3.25, p < 0.001].

### ALI onset

**Figure 2:**
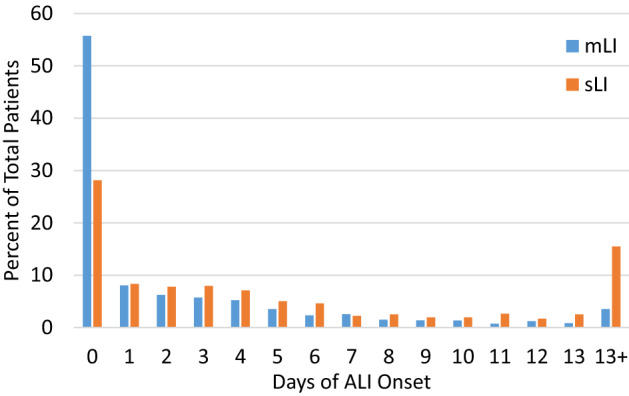
Histogram of mild and severe liver injury onset from days of hospitalization. Day zero represents admission date of patients. The average number of days patients developed mLI was 2.8 days and sLI was 6.7 days.

Figure 2 shows the histogram of organ injury onset in days during hospital admission. mLI and sLI patients developed liver injury on average 2.8 ± 7.4 and 6.7 ± 7.5 days during hospitalization, respectively. When combining both groups, ALI developed 3.6 ± 8.1 days on average during hospitalization.

**Figure 3:**
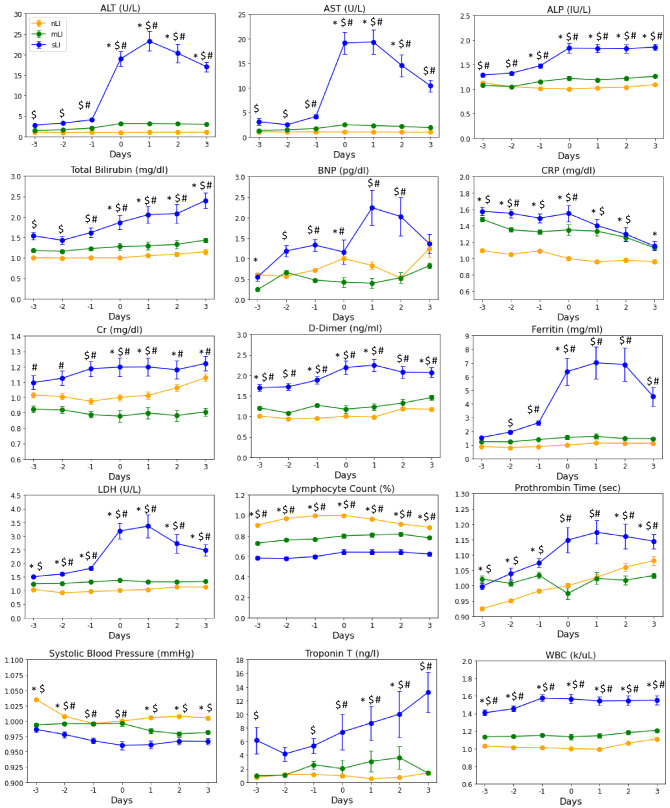
Temporal progression of laboratory tests, vitals and blood gases with time 0 representing day of liver injury onset in liver injury patients. No liver injury patient data was also centered around 3rd day after hospital admission. Values are normalized by dividing all data points by value of reading at time 0 of nLI group. *BNP* brain natriuretic peptide, *ALT* alanine aminotransferase, *AST* aspartate aminotransferase, *LDH* lactate dehydrogenase. No liver injury (nLI) is the orange line. mild liver injury (mLI) is the grey line, mild liver injury. Severe liver injury (sLI) is the blue line. Error bars are SEM. *Significant between nLI and mLI, ^$^significant between nLI and sLI, and ^#^significant between mLI and sLI based on linear mixed models.

Figure 3 depicts the time series of laboratory tests prior to and after ALI onset stratified by nLI, mLI, and sLI. Overall, sLI patients showed markedly worse laboratory abnormalities compared to mLI and nLI, whereas mLI patients showed similar patterns of laboratory test as nLI patients. ALT, AST, ferritin and LDH spiked at day of ALI onset and declined afterwards in sLI in marked contrast to those of mLI and nLI which remained lower and temporally invariant. ALP, bilirubin, BNP and DDIM were markedly elevated in sLI compared to mLI and nLI. PT in sLI patients was significantly higher than that of nLI patients on all days and between mLI and nLI on days 0 through 3. The reason for a trend of increasing PT value across time in the nLI group was unknown. There were 328 out of 718 (45.7%) of sLI patients had markedly elevated ALT readings (> 10 × UL N), suggestive of ischemic hepatitis. Vital signs (respiration and heart rate, systolic and diastolic blood pressure) were not plotted were similar across groups.

### Prediction of ALI onset

Using logistic regression, the top predictors of sLI onset were identified to be ALT, LDH, FERR, LYMPH. The AUCs of individual top predictors and all top predictors are shown in Table 2. AUCs were the highest on the day of ALI onset and dropped with days away from onset. The AUCs of the predictive model combining all top predictors were 0.78 ± 0.03, 0.86 ± 0.04, 0.91 ± 0.02 and 0.94 ± 0.02 on days − 3, − 2, − 1 and 0 respectively.

**Table 2:** Performance metrics (AUCs) of the individual top predictors of severe liver injury onset. For combined models using all top variables shown, AST was excluded due to its strong correlation with ALT.

	Day 0	Day − 1	Day − 2	Day − 3
ALT	0.99 ± 0.01	0.90 ± 0.03	0.83 ± 0.01	0.78 ± 0.02
LDH	0.75 ± 0.01	0.74 ± 0.02	0.73 ± 0.02	0.69 ± 0.01
FERR	0.70 ± 0.02	0.72 ± 0.02	0.72 ± 0.01	0.68 ± 0.03
LYMPH	0.61 ± 0.01	0.64 ± 0.01	0.67 ± 0.02	0.63 ± 0.02
Combined	0.94 ± 0.02	0.91 ± 0.02	0.86 ± 0.04	0.78 ± 0.03

### Recovery at hospital discharge

**Figure 4:**
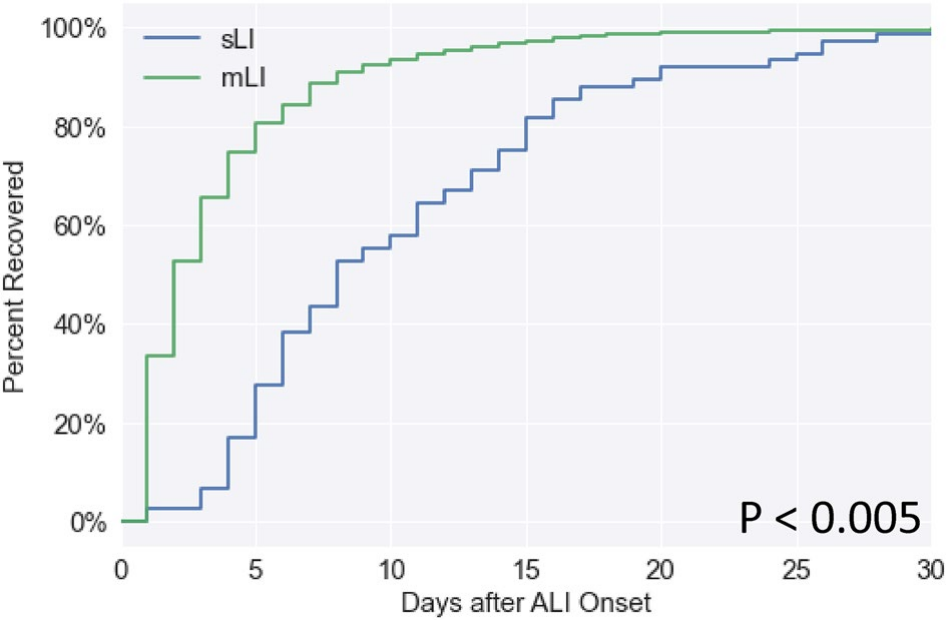
Kaplan–Meier curve for recovery of mild and severe liver injury relative to LI onset during hospitalization. Percent recovered on the vertical axis indicates those who recovered during hospitalization. Zero day indicates LI onset. The average number of days patients recovered from mLI was 3.8 and sLI was 10.4 days from LI onset (p < 0.005, log-rank test).

At hospital discharge, 15.7% of mLI and 23.4% of sLI patients (survivors) showed complete recovery defined by ALT dropping below an absolute threshold, and 21.7% of mLI and 66.0% of sLI patients showed at least partial recovery defined by 50% improvement of maximum ALT level (Table 3A). Figure 4 shows the Kaplan–Meier recovery curves for sLI and mLI after onset during hospitalization as defined by absolute threshold. Of patients who fully recovered, the average number of days to recovery for mLI was 3.8 ± 4.2 and sLI was 10.4 ± 6.7 days from ALI onset (p < 0.005).

**Table 3:** Number of mLI and SLI patients recovered (A) at hospital discharge and (B) post discharge defined by using absolute threshold and 50% improvement.

Recovery definition	Group	(A) At hospital discharge	(B) Post discharge
Absolute threshold	mLI	607/1812 (33.5%)	450/546 (82.4%)
	sLI	76/443 (17.2%)	112/146 (76.7%)
50% improvement	mLI	440/1812 (24.3%)	464/546 (85.0%)
	sLI	198/443 (44.7%)	139/146 (95.2%)

### Recovery at post discharge

This patient cohort returned to our hospital system post discharge 63 days (median, IQR 109) post discharge. 546 mLI (23.7%) and 146 sLI (20.3%) returned to our hospital system.

Of those returned, 82.4% of mLI and 76.7% of sLI patients post discharge showed complete recovery, and 85.0% of mLI and 95.2% of sLI patients showed at least partial recovery (Table 3B).

Table 4 shows patient characteristics and lab values of patients who did and did not recover from ALI by the absolute recovery definition upon return to the health system at 2 months. There were 130 (18.8%) patients who had not recovered; non-recovered patients were more likely to be male, Hispanic and had fewer co-morbid conditions. Those patients who did not recover were more likely to have had sLI as indicated by higher ALT, bilirubin, PT as well as sicker clinical course with lower SBP, higher CRP, FERR and WBC (p < 0.05). However, there was not a significant difference between the use of steroids, remdesivir, ICU stay or IMV.

**Table 4:** Characteristics of patients 2 months after hospital discharge from COVID-19 stratified by absolute recovery and non-recovery from ALI. Group comparisons (recovery vs non-recovery) of categorical variables were performed using the Chi-squared test. Group comparison of continuous variables in means and standard deviations (sd) were performed using the Wilcoxon test. A p-value < 0.05t was considered statistically significant.

	Not recovered	Recovered	p value
N (%)	130 (18.8%)	562 (81.2%)	
**Demographics**			
Age in years, mean (sd)	55.2 ± 16.9	61.4 ± 15.9	**< 0.001**
Female gender, n (%)	40 (30.8%)	254 (45.2%)	**0.003**
**Race/ethnicity, n (%)**			
Black/African American	29 (22.3%)	204 (36.3%)	**0.007**
White	9 (6.9%)	37 (6.6%)	0.77
Other	19 (14.6%)	76 (13.5%)	**0.02**
Hispanic	73 (56.2%)	245 (43.6%)	**0.01**
**Comorbidities, n (%)**			
Hypertension	42 (32.3%)	256 (45.6%)	**0.008**
Diabetes	45 (34.6%)	262 (46.6%)	**0.02**
Congestive heart failure	6 (4.6%)	26 (4.6%)	0.99
COPD	13 (10%)	76 (13.5%)	0.34
Coronary artery disease	8 (6.2%)	54 (9.6%)	0.28
Chronic kidney disease	45 (34.6%)	262 (46.6%)	**0.02**
Obesity	21 (16.2%)	109 (19.4%)	0.47
Invasive mechanical ventilation	1 (0.8%)	2 (0.4%)	0.99
ICU	23 (17.7%)	85 (15.1%)	0.55
Steroid	63 (48.5%)	279 (49.6%)	0.88
Remdesivir	25 (19.2%)	111 (19.8%)	0.99
**Return lab values, mean (sd)**			
Alkaline phosphatase, IU/L	139.99 (113.52)	97.87 (51.74)	**< 0.001**
Alanine aminotransferase, U/L	122.48 (358.23)	22.54 (9.28)	**< 0.001**
Aspartate aminotransferase, U/L	105.44 (378.71)	25.24 (14.14)	**< 0.001**
Bilirubin, mg/dL	0.77 (0.71)	0.6 (0.49)	**0.002**
Brain natriuretic peptide (pg/mL)	1543.93 (3495.06)	666.54 (2158.73)	0.09
Creatinine, mg/dL	1.35 (1.56)	1.54 (1.72)	0.29
C-reactive protein, mg/dL	10.15 (11.08)	4.75 (8.25)	**0.01**
d-dimer, µg/mL	3.45 (4.95)	1.93 (3.72)	0.11
Ferritin, ng/mL	721.83 (1379.59)	361.3 (430.63)	**0.03**
Lactate dehydrogenase, U/L	404.58 (418.27)	305.59 (174.64)	0.13
Lymphocytes, (%)	24.91 (14.64)	27.54 (12.62)	0.05
Prothrombin time, s	15.38 (3.63)	15.24 (7.62)	0.9
Systolic blood pressure, mmHg	129.82 (23.61)	135.19 (22.76)	**0.03**
Troponin-T, ng/mL	0.07 (0.18)	0.06 (0.42)	0.83
White blood cell count (k/µL)	9.08 (5.65)	8.01 (3.53)	**0.01**

Significant values are in bold.

Logistic regression was used to predict recovery at 2-month post discharge with clinical variables at hospital discharge. The top predictors were ALT, BNP, Cr and bilirubin values at hospital discharge. The prediction accuracy was 83.2% ± 2.2%.

## Discussion

This study identified clinical predictors of ALI onset during COVID-19 hospitalization and of ALI recovery at hospital discharge and 2 months post hospital discharge in an academic medical center in the Bronx. The major findings are: (i) acute liver injury is a common complication (39.9%) amongst hospitalized COVID-19 patients with male sex having higher susceptibility, (ii) COVID-19 patients develop ALI 3.6 ± 8.1 days on average after hospitalization, (iii) the adjusted mortality odds ratios are 4.15 and 1.69 for sLI and mLI respectively, (iv) the top predictors of sLI onset are ALT, LDH, FERR and LYMPH, and they accurately predict ALI onset a few days prior to onset, (v) only 33.5% of mLI and 17.1% of sLI patients completely recover at hospital discharge, (vi) most (76.7–82.4%) patients completely recover ~ 2 months post hospital discharge, with the clinical variables at discharge accurately predict recovery with 83.2% ± 2.2% accuracy ~ 2 months post hospital discharge.

### Incidence and mortality rate

The incidence rates of mLI and sLI in our study of 30.4% and 9.5% are consistent with current published data showing the ALI incidence among COVID-19 patients to range from 4 to 33%^11^ and 16–29%^23^. While our mortality OR were similar to other previously published studies including a recent meta-analysis, these studies had not separated mLI and sLI^23^. While other studies reported no association with ICU admission^26,27^ and mortality^26^, our study found that sLI was associated with higher rates of ICU admission and hospital mortality. One possible explanation is that our diverse cohorts consisted of primarily Black and Hispanic patients in the Bronx with significant disadvantaged socioeconomic status, suggesting potential health disparity. Health disparity in COVID-19 outcomes in generally has been reported previously^28^. Additionally, our data includes the initial phase of the pandemic in New York City, during which hospital resources and COVID-19 treatments were limited, resulting in comparatively worse outcomes including more severe illness and death. The wide ranges of incidence and outcome in the literature are not unexpected and could be due to data obtained from different phase of pandemic, geographic regions affected by the pandemic, cohort characteristics (i.e., demographics, hospitalization status, pre-existing conditions, and disease severity) as well as improving treatment for COVID-19 over time^29^.

We found male sex to be markedly more susceptible to ALI compared to females, consistent with a prior study reporting 61.7% of men had COVID-19 related ALI^30^. Although male sex increased susceptibility in general to COVID-19 disease severity and mortality have been reported previously^31^, male sex susceptibility to ALI in our study is among the high ends of the literature.

Interestingly, the sLI group was younger than nLI group and had fewer co-morbid conditions. This counterintuitive finding may reflect more aggressive care at times of limited resources including COVID-19 directed therapies and ICU level care. The sLI group was more likely to receive steroids and remdesivir, which may have resulted in hepatotoxicity. While our study only looked at use of steroids, remdesivir and IMV, there are several additional therapies including antibiotics which may result in drug-induced liver injury. ICU care suggests worse COVID-19 disease severity and risk of both direct COVID-19 related liver injury as well as ischemic injury.

Our model showed that ALT, LDH, FERR and lymphocytes are top predictors of sLI onset. Hypoxia was also a good predictor but not the top five. A possible explanation is that hypoxia was a general risk factor but not specific to sLI (i.e., low specificity). Although ALT was used as a definition of liver injury, ALT at the earlier time point (at discharge) was used to predict future (post discharge) outcome. LDH, FERR and lymphocytes, which are established markers of COVID-19 disease severity, are predictive of future sLI.

The degree of sLI was not limited to transaminase elevation; this subset of patients had significant increases in both bilirubin and PT, consistent with hepatic dysfunction. Of note, patients with sLI had higher Cr levels suggesting that along with ALI patients were susceptible to acute kidney injury. AKI is known to be a common complication of severe COVID-19 infection^32–35^.

### ALI onset

Our study analyzed graded liver injury and how clinical variables changed across time, which differs from prior studies that typically examined clinical variables at admission only. Longitudinal clinical variables were time-locked to the onset of liver injury in patients. Notably, sLI is associated with more severe COVID-19 disease, as indicated by how different the sLI time courses were compared to nLI and mLI patients. Physicians can intervene based on the laboratory tests that change early and slowly worsen over the time course of a patient because they can be a foreshadowing of liver injury, and thus, should closely monitor these laboratory tests in COVID-19 patients.

The top predictors of sLI onset were ALT, LDH, FERR and LYMPH, and they together accurately predict ALI onset. A previous study reported the top predictors of sLI to be ALT, LDH, respiration rate, ferritin, and lymphocyte, yielding an AUC of 0.88, 0.92, and 0.98 at − 2, − 1, and 0 days prior to onset, respectively^30^, which had a small sample size and consisted of mostly Caucasian patients. In contrast, our cohort which was larger and included a diverse population. ALT, ferritin, LDH and LYMPH were common between the two studies, suggesting they are likely predictive of sLI in COVID-19.

Through early identification of those patients at risk for sLI using these predictive values, physicians can monitor closely individuals during their hospitalization. The mechanisms underlying ALI in COVID-19 are likely multifactorial. Evidence of direct SARS-CoV-2 virus infection of hepatocytes via angiotensin-converting enzyme 2 (ACE2) receptors is sparse and controversial^11–13^. Systemic hypoxia, acute respiratory distress syndrome, hypotension, shock, or sepsis from COVID-19 complications, disproportional host-immune responses (such as inflammation, cytotropic and cytokine mediated immune responses), and hepatotoxicity from COVID-19 treatments (such as antiviral, antibacterial, steroid, anticoagulant, and immuno-modulatory medications) could also contribute to ALI in COVID-19 indirectly^14–19^.

### ALI recovery

Only a small percentage of ALI patients showed complete recovery at hospital discharge. sLI patients showed a markedly slower recovery time course leading up to discharge than mLI patients. These findings suggest that the majority of COVID-19 with ALI still had abnormal liver laboratory tests at the time of discharge and likely require follow up. Our results are in general agreement with a few studies that found that patients at 12 months after discharge had minimal elevation of liver enzyme and liver damage was mild and temporary and can return to normal within a short time during the recovery period^36,37^.

Our health system catchment captured a significant number of patients returning to our health system after COVID-19 hospitalization discharge, enabling longitudinal follow-up. In this cohort, the average patients returned to our hospital system 51 days (median) post discharge and most of these patients showed complete ALI recovery. Patients who returned to our hospitals were likely to have had more severe COVID-19 disease and/or have other major medical issues than those who did not. It is encouraging that most COVID-19 patients who developed ALI recovered completely ~ 2 months post hospital discharge, suggesting the ALI associated with COVID-19 is likely transient without long term damage and may be associated with hepatotoxicity from COVID-19 treatments (such as antiviral, antibacterial, steroid, anticoagulant, and immuno-modulatory medications). Characteristics of patients who are at risk for non-recovery at 2 months post-discharge include male sex and Hispanic ethnicity and had sLI with hyperbilirubinemia and coagulopathy.

### Limitations

This study has several limitations. First, although this retrospective study from a large hospital system of multiple hospitals in the Bronx offers real world clinical data on COVID-19 related liver injury and sequela, these findings need to be replicated in other institutions to increase generalizability. Our patient cohort is diverse, consisting of a large population of Black and Hispanic patients and our findings may not be generalizable to less diverse population. Additionally, the rapidly changing nature of the pandemic makes longitudinal comparisons between different waves of the pandemic challenging. Second, it is possible that some patients returned post discharge had a new event causing new ALI rather than residual injury associated with COVID-19. We only have data from patients who returned to our health system after hospital after discharge and we did not follow patients who did not return to our health system, which could result in bias toward patients with more post-acute COVID-19 symptoms and/or patients with more medical issues. Longer follow-up and prospective studies are warranted. Third, while patients with known liver diseases were excluded, there is the possibility that some patients included did have undiagnosed liver disease. Future studies should also examine the effect of COVID-19 on patients with chronic liver disease. Finally, as with any retrospective study, there could be unintended patient selection bias and unaccounted confounds.

## Conclusions

Acute liver injury is a common complication amongst hospitalized COVID-19 patients with male sex having higher susceptibility. While most patients did not completely recover from ALI at hospital discharge, but most patients with follow up data had recovered ~ 2 months post hospital discharge. Predictive models using readily available laboratory variables at discharge accurately predict ALI recovery status ~ 2 months post hospital discharge. A potential clinical implication is that heightened awareness for liver complications may be warranted when ALI is detected in COVID-19 patients. The ability to identify patients at-risk of persistent ALI early on would enable appropriate follow-up care including monitoring for the development of chronic liver disease.

## Data Availability

The datasets used are available from the corresponding author upon reasonable request.
